# The P450 oxidoreductase, RedA, controls development beyond the mound stage in *Dictyostelium discoideum*

**DOI:** 10.1186/1471-213X-8-8

**Published:** 2008-01-24

**Authors:** Daniela C Gonzalez-Kristeller, Layla Farage, Leonardo C Fiorini, William F Loomis, Aline M da Silva

**Affiliations:** 1 Departamento de Bioquímica, Instituto de Química, Universidade de São Paulo, Av. Prof. Lineu Prestes 748, 05508-000, São Paulo, Brasil; 2 Cell and Developmental Biology, Division of Biological Sciences, University of California San Diego, La Jolla, California, USA

## Abstract

**Background:**

NADPH-cytochrome-P450 oxidoreductase (CPR) is a ubiquitous enzyme that belongs to a family of diflavin oxidoreductases and is required for activity of the microsomal cytochrome-P450 monooxygenase system. CPR gene-disruption experiments have demonstrated that absence of this enzyme causes developmental defects both in mouse and insect.

**Results:**

Annotation of the sequenced genome of *D. discoideum *revealed the presence of three genes (*redA*, *redB *and *redC*) that encode putative members of the diflavin oxidoreductase protein family. *redA *transcripts are present during growth and early development but then decline, reaching undetectable levels after the mound stage. *redB *transcripts are present in the same levels during growth and development while *redC *expression was detected only in vegetative growing cells. We isolated a mutant strain of *Dictyostelium discoideum *following restriction enzyme-mediated integration (REMI) mutagenesis in which *redA *was disrupted. This mutant develops only to the mound stage and accumulates a bright yellow pigment. The mound-arrest phenotype is cell-autonomous suggesting that the defect occurs within the cells rather than in intercellular signaling.

**Conclusion:**

The developmental arrest due to disruption of *redA *implicates CPR in the metabolism of compounds that control cell differentiation.

## Background

NADPH-cytochrome-P450 oxidoreductase (CPR, EC 1.6.2.4) is a ubiquitous enzyme that is required for activity of the microsomal cytochrome-P450 (CYP) monooxygenase system [1,2]. This system is involved in the metabolic activation and/or detoxification of numerous foreign compounds as well as in the metabolism of endogenous substrates, such as steroids, alkaloids and fatty acids [3,4]. CPR belongs to a family of diflavin oxidoreductases which also includes the flavoprotein subunit of bacterial sulfite reductase (SiR) as well as a methionine synthase reductase and the cytoplasmic NADPH-dependent diflavin oxidoreductase 1 (NDOR1) identified in eukaryotic cells [5-8]. In addition, the diflavin reductase domain is found in fusion with cytochromes P-450 or with hemoprotein forming complex multidomain enzymes such as the cytochromes P450BM3 and the nitric oxide synthases [6].

CPR is a membrane anchored ~78 kDa enzyme which contains one molecule each of FAD and FMN bound as prosthetic groups that facilitate transfer of electrons of NADPH to the prosthetic heme group of CYP [1,2,9]. CPR is also involved in transferring electrons to other molecules, including heme oxygenase, squalene epoxidase and cytochrome b_5 _[10-12].

Despite the diversity of CYP isoforms that can be found in a single species [13], CPR in most organisms, except in certain plants and some zygomycetes, is encoded by only one gene [14-23]. Inactivation of the single-copy CPR gene in *Saccharomyces cerevisiae *results in mutants that accumulate only 25% as much ergosterol as observed in wild-type strains which probably accounts for the increased sensitivity of these mutants to the antifungal drug ketoconazole [16,24]. Moreover, it has been reported that cytochrome b_5 _gene can suppress the phenotype resulting from disruption of the CPR gene and therefore might function as an alternative electron donor for CYP activity in yeast [25-27]. In the fungus *Gibberella fujikuroi *loss of CPR leads to a reduced growth rate and has a strong influence on gibberellin biosynthesis [20].

CPR gene-disruption experiments in mouse have demonstrated that absence of this enzyme causes defects leading to mid-gestational lethality [28-30]. *In situ *hybridization studies have shown high levels of CPR expression in mesenchymal cells of the limbs and developing olfactory neuroepithelia [31]. CPR has been implicated in odorant clearance in insect antennae [17] and in ecdysone 20-hydroxylation during insect embryonic development [32].

Annotation of the sequenced genome of *D. discoideum *[33] revealed the presence of three genes that encode putative members of the diflavin oxidoreductase protein family. DDB0187719 (*redC*) on chromosome 5 encodes a polypeptide of 633 amino acids, which is 56% similar to human NADPH-dependent diflavin oxidoreductase 1 (NDOR1), a cytoplasmic enzyme highly expressed in cancer cell lines with as yet unknown functions [8]. Two genes, *redA *(DDB0215407) on chromosome 6 and *redB *(DDB0190667) on chromosome 1, show about 50% similarity to CPR proteins in humans, rats, *Drosophila *and yeast. We found that inactivation of *redA *in *Dictyostelium *results in developmental arrest at the mound stage.

## Results and Discussion

### Identification of the disrupted gene in the REMI mutant *redA*^-^

**Figure 1:**
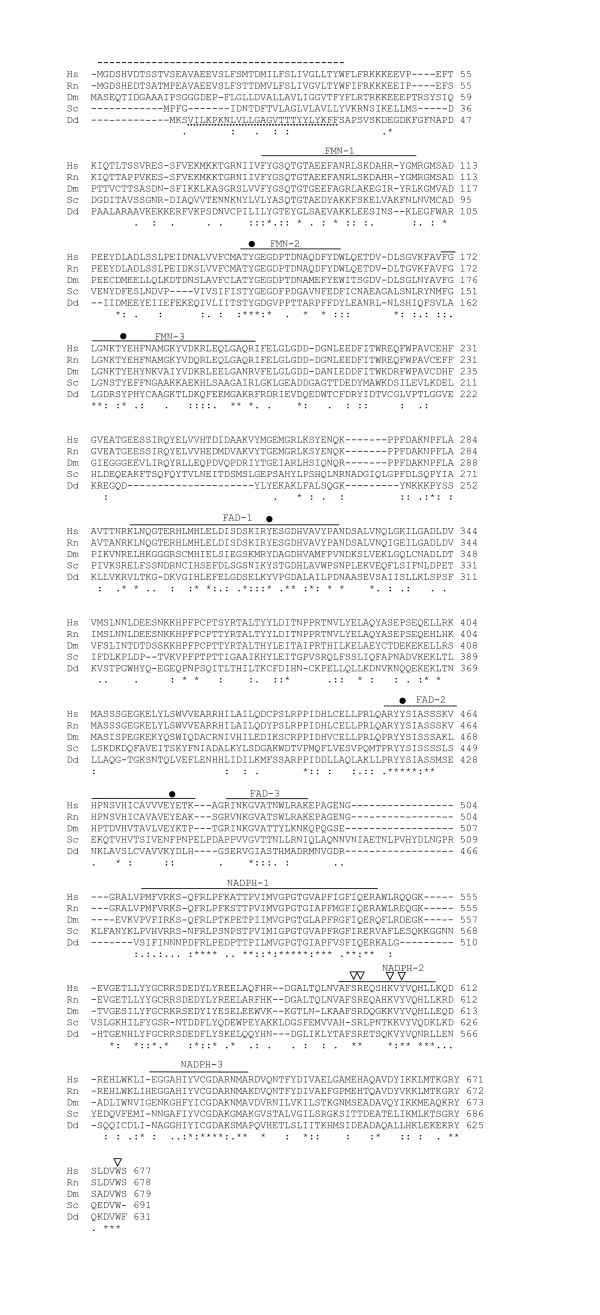
**Alignment of CPR aminoacid sequences**. Comparison of *Dictyostelium discoideum *(Dd) NADPH cytochrome P450 oxidoreductase (RedA) aminoacid sequence with orthologs from *Homo sapiens *(Hs, P16435), *Rattus norvegicus *(Rn, P00388), *Drosophila melanogaster *(Dm, CAA63639) and *Saccharomyces cerevisiae *(Sc, P16603) using CLUSTAL W program. Identical and conserved amino acids are denoted with (*) and (:), respectively. Semi-conservative changes are indicated with a single dot. Binding domains for FMN, FAD and NADPH are indicated. Closed circles indicate highly conserved aromatic amino-acid residues that are particularly important in flavin binding. Open triangle points residues involved in NADPH discrimination. The ~56 aminoacid and the ~20 aminoacid hydrophobic segments at the N-terminal are indicated by dashed line in human CPR and dotted line in *Dictyostelium *CPR, respectively.

The mutant described in this work was isolated from a REMI-mutagenic library screen for morphological mutants of *Dictyostelium discoideum*. Strain DG1047 was picked because it forms yellow mounds that fail to make proper fruiting bodies. A portion of the disrupted gene was isolated from this strain by plasmid rescue in *E. coli *[34]. This fragment was used to screen a cDNA library prepared from vegetative cells. The largest cDNA insert (2094 bp) was sequenced and found to encode a putative protein of 631 amino acids with ~50% similarity to CPRs from human, rat, *Drosophila*, and yeast (Figure 1). The gene was designated *redA *as a mnemonic that it is likely to act in a redox reaction.

The *D. discoideum *CPR encoded by *redA *shows considerable conservation in the regions proposed to be involved in binding FMN, FAD and NADPH [2,9,18,35-37]. It is worth mentioning that the NADPH-2 region pointed out in Figure 1 contains the three residues Ser-596, Arg-597 and Lys-602 (positions numbered according human CPR) involved in the binding of the enzyme to the 2' phosphate of NADPH [2,38]. In addition, the conserved carboxi terminal motif G/K/N-R-Y-x-x-D-V/T-W is present in *D. discoideum *CPR. It has been demonstrated that the tryptophan in this motif plays a major role in discrimination of NADPH [36].

Analysis of the predicted RedA amino acid sequence by Signal P and TMHMM programs [39,40] revealed a N-terminal hydrophobic segment of ~20–25 amino acids (Figure 1) that should be sufficient for its anchorage to a membrane. Despite the fact that the hydrophobic N-terminal of mammalian CPRs is approximately 56 amino acids long [2], in plants and in fungi a shorter hydrophobic N-terminal is sufficient for membrane interaction [24,41-43]. Moreover, it has been proposed that interaction of human CPR to membranes and to CYPs is likely to involve additional hydrophobic patches on CPR surface [2,44].

Southern blot analysis of *D. discoideum *genomic DNA cleaved with a variety of restriction enzymes showed that RedA is encoded by a single copy gene (data not shown). Comparison of *redA *cDNA sequence with the *Dictyostelium *genome sequence [33] confirmed this result and showed that *redA *is an intronless gene located on chromosome 6. In most organisms analyzed, such as humans, mouse, *Drosophila*, *S. cerevisiae *and filamentous fungi, the CPR gene is present as a single copy and in the fruit fly two alternative splicing isoforms have been identified [14-18,20,45]. On the other hand, plants and certain fungi often have multiple copies of CPR gene [21,22,41,43,46,47]. We found two CPR genes in the *Dictyostelium *genome, *redA *and *redB*. Even though the amino acid sequences of *redA *and *redB *are 52% similar to each other, their nucleotide sequences are highly diverged and the genes appear to have evolved independently for a long time. *D. discoideum *genome has a third gene (*redC*) that encodes an additional member of diflavin oxidoreductases family which conserves sequences defined as binding domains for FMN, FAD and NADPH but lacks the N-terminal hydrophobic region found in RedA and RedB.

### Expression of *redA *during growth and development

**Figure 2:**
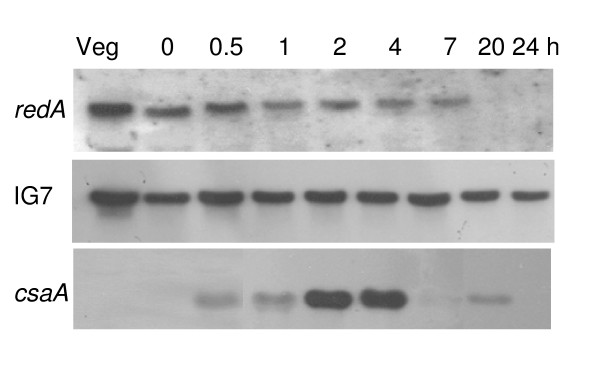
**Expression of *redA *during growth and development of wild type AX4 cells**. Exponentially growing AX4 cells (Veg) were starved on filter pads and harvested at the indicated times (h) after starvation. Identical Northern blots of total RNA samples were probed with *redA*, IG7 and *csaA *cDNAs as indicated. IG7 transcript is expressed at similar levels throughout the *D. discoideum *development [59].

**Figure 3:**
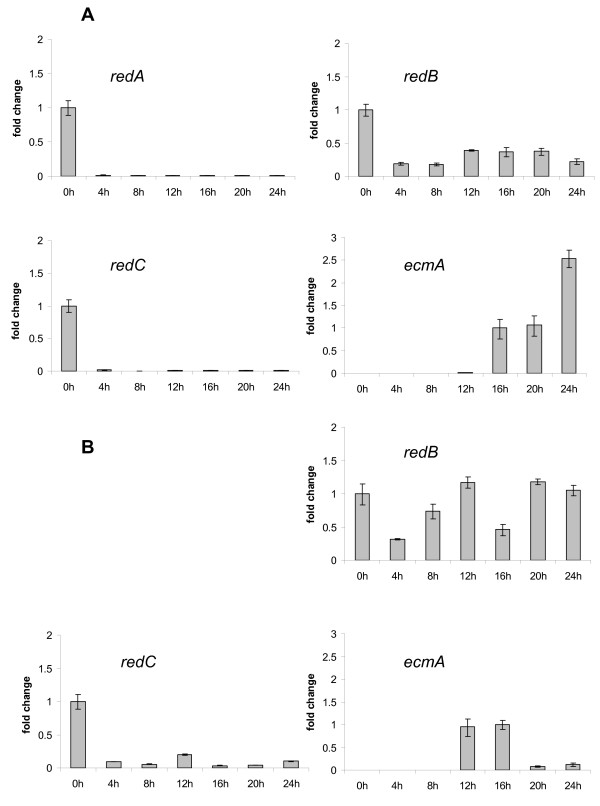
**Transcriptional profile of *redA*, *redB*, *redC *and *ecmA***. Exponentially growing AX4 **(A) **and *redA*^- ^**(B) **cells were starved on filter pads and harvested at the indicated times (h) after starvation. Transcript levels for *redA*, *redB *and *redC *genes are relative to 0 h cells. Fold change for *ecmA *are relative to transcript levels detected at 16 h. Error bars represent the standard deviation from two independent experiments where qPCR assays were performed in triplicate.

The expression of *redA *was monitored by Northern blot using a *redA *cDNA fragment as probe. As shown in Figure 2, a single mRNA species of 2.3 kbp was present in growing cells and decreased in abundance upon starvation of the cells on filter pads. No *redA *mRNA could be detected late in development (Figure 2 and 3A) in agreement with the *redA *expression profile determined on microarrays [48]. As a control, we probed for the *csaA *mRNA encoding the cell adhesion protein gp80 which is highly expressed during early aggregation [49]. This mRNA accumulated rapidly to reach peak levels by 2 hours and decreased after 4 hours of development (Figure 2).

In contrast, we found that *redB *is constitutively expressed throughout development of AX4 cells, while *redC *is expressed at detectable levels only in vegetative growing cells being repressed upon cell starvation. As a late development marker we monitored expression of *ecmA *[50], a prestalk-specific gene (Figure 3A).

CPR mRNA has distinct expression patterns during the development of several tissues and organs in mice, and this expression is not coordinated with expression of CYP genes [29,31,51,52]. The CPR gene is expressed in the early stages of embryonic development, suggesting that CPR-dependent processes may be important at this stage of the embryogenesis [28,31,32,53]. In *Drosophila melanogaster *the CPR gene shows high levels of expression in various embryonic tissues as well as in the antenna of adults [17]. In parsley and *Arabidopsis *there are two CPR genes, one of which is constitutively expressed while the other is induced by biotic and abiotic stresses [21,47].

### Phenotype of the *redA *minus mutants

**Figure 4:**
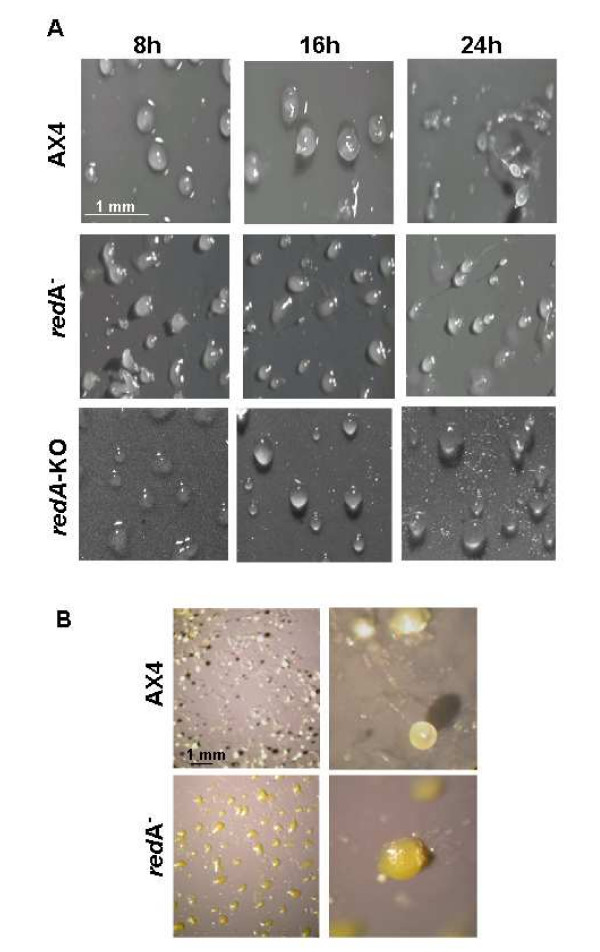
**Disruption of *redA *impairs development at mound stage**. **(A) **Exponentially growing AX4 wild type cells and the mutants *redA*^- ^and *redA*-KO were starved on filter pads and photographed at the indicated times (h) after starvation. **(B) **AX4 fruiting bodies and redA^- ^yellow mounds after 48 hours starvation on filter pads are shown at lower (left) and at 5× higher magnification (right).

To confirm that the *redA*^- ^phenotype is due to the disruption of *redA*, we generated new mutant strains by homologous recombination with the original plasmid pRED isolated from *redA *REMI mutant. Effective *redA *disruption was checked by Southern blot analyses of genomic DNA from blasticidin-resistant clones (data not shown) and six independent knockout clones were isolated which showed the same mound-arrest phenotype. One strain (*redA*-KO) was selected for further analyses. As shown in Figure 4A, when compared to wild type AX4 strain, the majority of cells of *redA*^- ^and *redA*-KO mutants failed to make mature fruiting bodies after 24 h development on filters, and was arrested at the mound stage where they accumulated a yellow pigment (Figure 4B). It should be pointed out that the *redA*-KO mutants form a few tipped aggregates in the mound population after 48 hours starvation (data not shown). On the other hand, the original REMI and the recapitulated mutants did not show any significant differences in their growth curves when compared to the wild type AX4 (data not shown).

**Figure 5:**
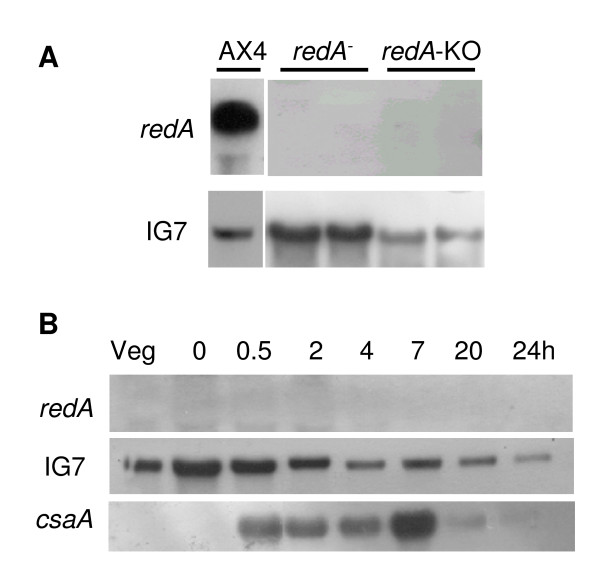
**Disruption of *redA *results in cells lacking *redA *transcript**. **(A) **Total RNA was prepared from exponentially growing AX4 cells (AX4) and from two independent clones of the mutants *redA*^- ^and *redA*-KO. Identical Northern blots were probed with *redA *and IG7 cDNAs as indicated. **(B) **Exponentially growing *redA*^- ^cells (Veg) were starved on filter pads and harvested at the indicated times (h) after starvation. Identical Northern blots of total RNA samples were probed with *redA*, IG7 and *csaA *cDNAs as indicated.

As expected, *redA*^- ^and *redA*-KO mutants did not express *redA *mRNA (Figure 5A). Despite their developmental defect, *redA*^- ^cells expressed *csaA *during development on filter pads (Figure 5B). Moreover, *redC *transcriptional profile is reasonably similar in wild type AX4 and in *redA*^-^cells (Figure 3) as its transcript levels strongly decrease upon starvation. On the other hand despite being expressed throughout development both in AX4 and *redA*^- ^cells, *redB *transcript accumulates at higher levels in the latter (Figure 3). Also the peak of expression of *ecmA *was found to be advanced by four hours in the *redA*^- ^strain as compared to the wild type (Figure 3).

**Figure 6:**
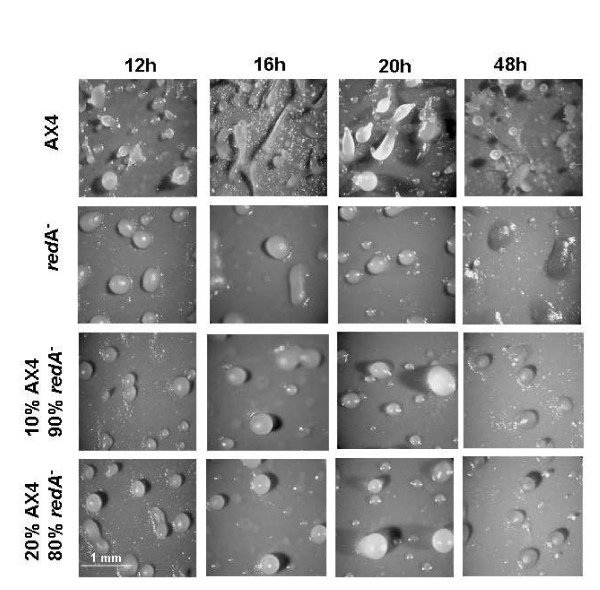
**AX4 cells do not rescue *redA*^- ^phenotype**. Exponentially growing *redA*^- ^and AX4 wild type cells were starved on filter pads mixed at the indicated proportions. At the indicated times (h) after starvation cells were photographed.

The developmental defect of *redA*^- ^mutant is not rescued by mixing with AX4 wild type cells. As shown in Figure 6, mixing 10% or 20% of AX4 with *redA*^- ^mutant did not overcome the mutant mound arrest indicating autonomy of the mutant phenotype. Moreover, the mutant cells did not inhibit wild type cells from forming fruiting bodies when they were developed together in equal numbers (not shown).

**Figure 7:**
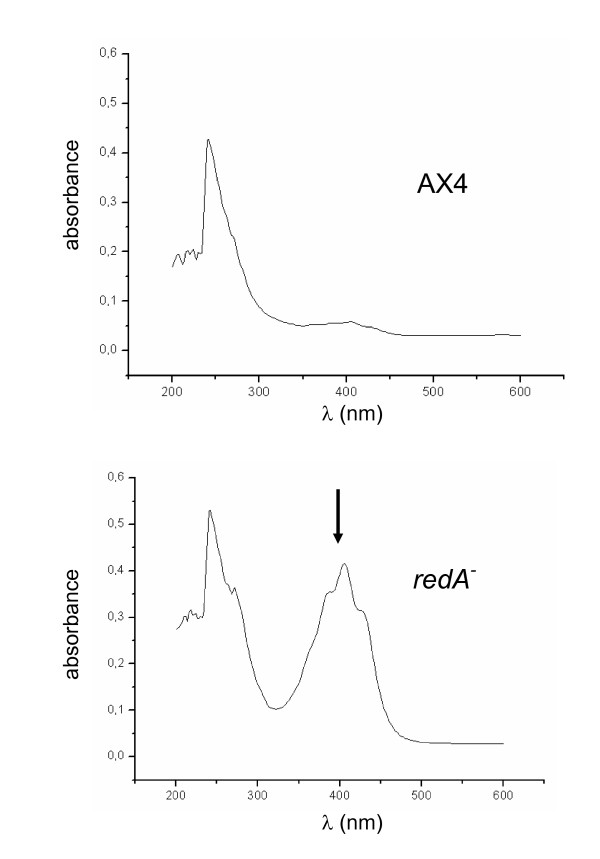
**UV and visible spectra of the chloroform extracts from AX4 and *redA***^-^. Chloroform extracts of AX4 and *redA*^- ^cells collected after 48 hours starvation were analyzed using a UV/Visible spectrophotometer. The arrow points the absorption peak at 400 nm observed in the *redA*^- ^cell extract.

As mentioned above both *redA*^- ^and *redA*-KO mutants form yellow mounds upon starvation (Figure 4B). We have observed that after 48 hours filter starvation the mounds and even the filter turn a strong yellow colour. This does not reflect premature spore formation since the yellow mounds do not contain any viable spores (data not shown). Chloroform extracts of *redA*^- ^mounds collected after 48 hours starvation show an absorption peak at 400 nm which is not observed in AX4 cells (Figure 7). We did not succeed in characterizing the metabolites that accumulate in redA-mutants despite many attempts.

CPR is a key enzyme in many metabolic processes, as a consequence of its close interactions with cytochrome P450 heme oxygenases. In particular, its participation in synthesis and/or degradation of important cellular compounds, such as retinoic acid, cholesterol and steroid hormones [2,29,30,35,36,54] may be related to abnormalities observed in development of organisms where CPR expression is abolished [28,32,55]. Development of homozygous mouse embryos carrying inactivating mutations in both alleles of the CPR gene is severely impaired, but lethality is only observed 10 to 13 days after zygote formation [28,29]. These findings indicate the importance of CPR in early animal development. Our results point to a role for a *D. discoideum *CPR in the metabolism of factors which control its cellular differentiation.

## Conclusion

The *D. discoideum *genome encodes three genes (*redA*, *redB *and *redC*) for enzymes of diflavin oxidoreductases family. Disruption of *redA *led to mutant cells that form yellow mounds that fail to make proper fruiting bodies. The developmental arrest shown by this mutant implicates *redA*-encoded P450 oxidoreductase in the metabolism of compounds that control cell differentiation.

## Methods

### Cells and culture conditions

*Dictyostelium discoideum *strain AX4 and derived transformants were grown in axenic medium (HL-5) or in SM agar plates on lawns of *Klebsiella aerogenes *[56]. Complete and synchronous developmental program was undertaken by washing cells with 20 mM phosphate buffer (pH 6.4) and depositing them at 5 × 10^7 ^on nitrocellulose filters supported on buffer-saturated pads as previously described [56]. Strain DG1047 (*redA*^-^) was selected from a *Hind*III REMI-mutagenised library of strain AX4 selected for integration of the pBSR3 vector which carries the blasticidin S resistance cassette [34,57]. Morphological mutants were recognized by the structures formed within plaques generated by the blasticidin-resistant cells grown on SM agar in association with *K. aerogenes*.

### Cloning of *Dictyostelium redA *cDNA

Regions flanking the plasmid insertion site in the REMI-mutant were isolated by plasmid rescue as described [34]. Genomic DNA from *redA*^- ^strain was digested with *Hind*III ligated and electroporated into *Escherichia coli *SURE cells (Stratagene). The rescue plasmid pRED was isolated from the ampicillin-resistant bacterial transformants and sequenced. A 2190 bp sequence partially encoding *redA *gene has been deposited in GenBank (access number AF012946). *Hind*III-linearized pRED was used to recapitulate *redA *mutation by homologous recombination as described [34].

A 1.8 kbp genomic fragment obtained from pRED by digestion with *Hind*III and *Sma*I was used as probe to screen a Lambda-ZAP (Stratagene) cDNA library derived from AX4 *D. discoideum *vegetative cells (kindly provided by Dr. Hudson Freeze, The Burnham Institute, La Jolla, USA). Screening of 200,000 plaques under high stringency conditions yielded twenty positives clones that were subjected to *in vivo *excision from a phagemid by transformation of *Escherichia coli *XL-1 blue MRF' (Stratagene). The pBluescript SK clone (2B) with the largest cDNA insert was completely sequenced on both strands and the sequence was deposited in GenBank (access number DQ344637).

Molecular cloning procedures were essentially as described [58], unless otherwise noted. DNA sequencing was performed on an ABI 377 automated sequencer (Perkin-Elmer).

### Northern Blots

Total RNA was isolated from 5 × 10^7 ^*D. discoideum *cells at various developmental stages by using the Trizol (Invitrogen). Formaldehyde-agarose gel electrophoresis of RNA (20 μg) and transfers to nylon membranes (Amersham), were performed as described [58]. Probes were prepared with gel-purified DNA fragments radiolabeled with [α-^32^P]dATP and [α-^32^P]dCTP by the random hexanucleotide priming method (Random Primers DNA Labeling System, Invitrogen).

### RT-qPCR

Reverse transcription was carried out with 5 μg of *D. discoideum *total RNA primed with a mixture of oligo dT and random hexamers using SuperScript First-Strand Synthesis System (Invitrogen). A 20 μg amount of the resulting cDNA were subjected to quantitative PCR using Platinum SYBR Green qPCR SuperMix UDG (Invitrogen) on a GeneAmp 5700 System (Applied Biosystems) using the default thermocycler program for all genes. Threshold values were normalized according to C_t _of *D. discoideum *mitochondrial large subunit rRNA (IG7), which is expressed at similar levels throughout the *D. discoideum *development [59]. The fold change of each gene was calculated using the 2^-ΔCt ^method [60]. qPCR assays were performed in triplicate with the following gene-specific primer pairs: *redA *(5'-CCTATGGTGATGGTGTTCCACCAAC-3' and 5'-CCCCACTAAATTGAATATGTGAAAGATTTAAACGA-3'), *redB *(5'-GCAACCGAAGAAGCAAACGAAGAATACAAT-3' and 5'-CAAAGGTTGAAGACCTGGGAAAGATTCTAA-3'), *redC *(5'-AGGTGGAGTCTTTGAAAGATGTTGTAAAAATCC-3' and 5'-GGTCCAGGTACTGGTGTTGCAC-3'), *ecmA *(5'-AGCTGATAGTTGCGATTCCA-3' and 5'-TACCTCCTGTACCACCACCA-3') and *rnlA *(IG7) (5'-GTGGTTCGGCACCTCGAT-3' and 5'-CACCCCAACCCTTGGAAACT-3').

### Chloroform extraction

5 × 10^8 ^AX4 and *redA*^- ^cells developed on filters for 48 hours were extracted with 1 ml chloroform and the organic phase was collected by centrifugation at 3000 × g for 5 min at 4°C. UV/Visible spectra of the chloroform extracts were obtained in UV-2401PC Shimadzu spectrophotometer.

### Sporulation efficiency assay

Mutants and wild-type cells were allowed to develop on nitrocellulose filters. At 0, 8, 12, 16, 20, 24 and 48 hours the cells were harvested from the filters with 20 mM phosphate buffer (pH 6.4) and sporulation efficiency was determined by detergent and heating treatment of the cells following plating on SM agar in association with *K. aerogenes *[56]. The number of plaques in the bacterial lawn indicated the number of viable spores. Wild-type AX4 cells submitted to 5-day starvation were used as a positive control for these experiments, to ensure recovery of fruiting bodies with viable spores.

## List of abbreviations used

CPR, NADPH-cytochrome P450 oxidoreductase; FAD, flavin adenine dinucleotide; FMN, flavin mononucleotide.

## Authors' contributions

DCGK carried out most of the experimental work and drafted the manuscript. LF performed RT-qPCR assays and helped with chloroform extractions. LCF was involved in the initial steps of this work and helped with cDNA cloning and sequence analyses. WFL isolated strain DG1047, participated in the design of the study and in writing the manuscript. AMDS coordinated the study, participated in its design and wrote the manuscript. All authors read and approved the final manuscript.
